# Do Nonnutritive Sweeteners Alter Appetite and Food Preferences in Children? Current Evidence and Future Directions

**DOI:** 10.3390/nu18203390

**Published:** 2026-10-16

**Authors:** Agnieszka Kowalczyk, Julia Tworowska

**Affiliations:** Department of Pediatrics, Allergology and Gastroenterology Collegium Medicum, Bydgoszcz Campus, Nicolaus Copernicus University, Torun, M. Curie Skłodowskiej 9 Street, 85-094 Bydgoszcz, Poland; j.tworowska@cm.umk.pl

**Keywords:** nonnutritive sweeteners, pediatric nutrition, children, appetite, energy intake, sweet taste preference, food choice, dietary behavior

## Abstract

**Background/Objectives**: Nonnutritive sweeteners (NNS) are increasingly consumed by children, but their effects on appetite regulation and food preference remain uncertain. This review evaluated whether NNS influence appetite, satiety, subsequent energy intake, sweet taste liking, preferred sweetness, or food choice in children, and how key study and developmental factors affect interpretation. **Methods**: This narrative review was informed by a structured PubMed search from database inception through August 2026, limited to English-language articles. Pediatric randomized and controlled studies were prioritized for direct behavioral outcomes; observational studies characterized habitual exposure and longer-term associations. **Results**: Direct pediatric evidence is limited and heterogeneous. Available studies do not show a consistent effect of NNS on subjective appetite or satiety. Differences in subsequent energy intake are most consistent with caloric substitution and incomplete compensation when NNS replace sugars, rather than an intrinsic appetite-suppressing effect. Pediatric studies show that NNS-sweetened products can be accepted and liked and that flavor–energy associations may influence learned liking, but they do not establish that sustained NNS exposure alters generalized preferred sweetness or habitual food choice. Anthropometric findings are indirect and depend strongly on comparator and study design. Interpretation is further influenced by sweetener type, dose, food matrix, exposure duration, and developmental stage. **Conclusions**: Current evidence does not demonstrate a consistent pediatric behavioral effect of NNS on appetite, preferred sweetness, or food choice. NNS may reduce sugar and energy intake when replacing sugar-sweetened products, but long-term developmental effects remain uncertain. Pediatric interventions directly measuring preferred sweetness and subsequent food choice are needed.

## 1. Introduction

High intake of free sugars, particularly from sugar-sweetened beverages, is associated with unhealthy weight gain and dental caries in children and adolescents. Current dietary guidance therefore recommends limiting free-sugar intake and identifies water and unsweetened beverages as the preferred choices for children [1,2]. Dietary habits established early in life may persist into later childhood; for example, sugar-sweetened beverage intake during infancy has been associated with higher consumption at 6 years of age [3]. Reducing exposure to highly sweetened foods and beverages may therefore be particularly relevant during the period when dietary habits and food preferences are developing.

Nonnutritive sweeteners (NNS) are increasingly used in food reformulation to reduce added sugars while retaining sweetness. They comprise a heterogeneous group of compounds that provide intense sweetness with little or no energy, including aspartame, sucralose, saccharin, acesulfame K, and steviol glycosides [4]. Exposure is already common in children. Low-calorie sweetener consumption has been documented in US and UK pediatric populations [5,6], and a recent study of Chilean 3-year-olds found that 76% consumed at least one NNS [7]. NNS are consumed from multiple dietary sources, and parents may not always recognize their presence in products marketed to young children [5,7,8]. Consequently, reducing dietary sugar does not necessarily reduce exposure to an intensely sweet taste. For consistency, the term NNS is used throughout this review; the term non-sugar sweeteners (NSS) is retained only when referring specifically to terminology used by the World Health Organization (WHO).

The use of NNS raises several related but distinct questions. First, does replacing caloric sugars with NNS reduce energy intake primarily through removal of sugar-derived energy, or do NNS themselves influence appetite and satiety? Second, does repeated exposure to sweetness in the absence of an equivalent caloric load alter the preferred level of sweetness or learned food preferences? Third, does such exposure influence subsequent food or beverage choices in children? These questions should not be treated as interchangeable. Subjective appetite, satiety, subsequent energy intake, sweet taste liking, food choice, and body-weight outcomes represent different constructs and may reflect different underlying mechanisms.

Sustained intervention studies generally show lower energy intake or less weight gain when NNS replace caloric sugars, although the magnitude of the effect depends strongly on the comparator [9,10,11]. Trials comparing NNS-containing products with sugar-sweetened alternatives primarily evaluate the consequences of caloric substitution, whereas comparisons with water or unsweetened products are more informative about effects attributable to NNS themselves [9,10]. At the same time, sweet taste contributes to sensory signaling and food reward, while post-ingestive nutrient signals participate in learned associations between the sensory properties of food and its metabolic consequences [12,13,14,15,16]. Experimental and preclinical work has therefore raised the possibility that repeated dissociation between sweetness and energy delivery could influence appetite regulation, energy compensation, or learned preferences [17,18,19]. Whether these mechanisms translate into clinically relevant behavioral effects in children remains uncertain [4,11,20].

The pediatric literature is particularly difficult to interpret because NNS should not be considered a biologically uniform exposure. Individual sweeteners differ in physicochemical properties, absorption, metabolism, gastrointestinal exposure, and sweetness intensity, while studies vary in the compounds examined, dose, food matrix, duration of exposure, and comparator [4]. Study design further affects interpretation. Observational studies pose additional problems, particularly reverse causality and residual confounding, because NNS consumption may itself be influenced by baseline adiposity, dietary patterns, or attempts to reduce sugar intake [9,11]. Moreover, pediatric studies have focused predominantly on exposure prevalence, body weight, and metabolic outcomes, with relatively few directly assessing appetite, satiety, spontaneous energy intake, sweet liking, preferred sweetness or subsequent food choice [4,9,11,20].

Extrapolation from adult or preclinical studies also requires caution. Children and adolescents differ from adults in growth, energy requirements, pubertal development, dietary autonomy, and the process through which food preferences are learned. Mechanistic evidence from animal models and adult studies may therefore establish biological plausibility but cannot by itself demonstrate that the same effects occur in children. The 2023 WHO guideline recommends against the use of non-sugar sweeteners as a strategy for long-term weight control or reduction in noncommunicable diseases; this is a conditional recommendation reflecting limitations in the available evidence [21]. This public-health recommendation is distinct from toxicological assessments used to establish safe intake levels for individual sweeteners [21]. Previous systematic reviews and meta-analyses have provided important syntheses of NNS in relation to body weight, metabolic outcomes, and broader health effects [9,10,11]. The present review addresses a different but complementary question: what evidence directly supports an effect of NNS on eating-related behavior during childhood? We therefore distinguish direct pediatric evidence on appetite, satiety, subsequent energy intake, sweet taste preference, and food choice from indirect anthropometric and dietary evidence, and from mechanistic findings derived predominantly from adult and preclinical studies. Particular attention is given to the comparator, specific sweetener, dose, food matrix, duration of exposure, and developmental stage as potential sources of heterogeneity.

Accordingly, this review addresses four questions:

1. Do NNS acutely affect appetite, satiety, or subsequent energy intake in children compared with caloric sugars or unsweetened comparators?
2. Does repeated exposure to NNS alter sweet taste liking or the preferred level of sweetness during childhood?
3. Does NNS exposure influence subsequent food or beverage choices in children?
4. How do sweetener type, dose, food matrix, exposure duration, comparator, and developmental stage influence the interpretation of these outcomes?

Body weight, adiposity, early-life exposure, and metabolic outcomes are considered where they provide relevant developmental or clinical context, but they are not treated as equivalent to direct measures of appetite or food preference.

## 2. Methods

This narrative review was informed by a structured literature search conducted in PubMed from database inception through August 2026. No restrictions on publication date were applied. Searches were limited to articles published in English and used combinations of terms including “nonnutritive sweetener”, “non-sugar sweetener”, and “artificial sweetener” together with “child”, “adolescent”, “appetite”, and “sweet taste”. Reference lists of relevant systematic reviews and meta-analyses were also examined to identify additional primary studies that were not retrieved directly through the database searches.

The review focused primarily on studies conducted in children and adolescents. Pediatric randomized and controlled intervention studies were prioritized when evaluating direct effects on appetite, satiety, energy intake, sweet taste preference, and food choice, whereas observational studies were used to characterize habitual exposure, dietary patterns, and longer-term associations. Studies conducted in adults and experimental animal models were included only where they provided mechanistic context relevant to sweet taste perception, post-ingestive nutrient signaling, gut–brain communication, food reward, or learned preference. These mechanistic data were not considered equivalent to direct pediatric evidence.

Because the aim was to provide a narrative and conceptually integrated synthesis rather than a formal systematic review, no prespecified exclusion criteria or formal risk-of-bias assessment were applied. Evidence was interpreted according to study design, population, comparator, duration of exposure, sweetener type, and outcome assessed. Particular attention was given to distinguishing studies evaluating replacement of caloric sugars with NNS from studies comparing NNS with water or unsweetened products, as these designs address different causal questions. When several systematic reviews included the same primary studies, overlapping studies were considered as a single underlying body of evidence rather than as independent confirmation of an effect. Multiple publications arising from the same underlying trial were treated as a single study population and were considered separately only when they reported distinct outcomes relevant to the research questions.

## 3. Nonnutritive Sweeteners: Definition, Types and Exposure in Children

NNS comprise a heterogeneous group of compounds used to provide sweetness while contributing little or no energy at the amounts typically consumed. Commonly used NNS include aspartame, sucralose, saccharin, acesulfame K, and steviol glycosides [4,21]. These compounds differ substantially in chemical structure, absorption, metabolism, and gastrointestinal exposure [4,22]. Such differences are relevant when interpreting experimental and clinical studies, because findings obtained with one sweetener cannot necessarily be generalized to other NNS. Selected characteristics of commonly used NNS are summarized in Table 1.

Terminology is not uniform across the literature. Terms such as nonnutritive sweeteners (NNS), non-sugar sweeteners (NSS), and low-calorie sweeteners (LCS) are often used with partially overlapping meanings. The WHO uses the term NSS to describe synthetic and naturally occurring or modified nonnutritive sweeteners that are not classified as sugars [21]. Its definition explicitly excludes low-calorie sugars and sugar alcohols (polyols), such as erythritol, xylitol, and sorbitol, because these compounds are caloric sugar derivatives and are metabolically distinct from high-intensity sweeteners [21]. For consistency, the term NNS is used throughout this review; polyols are excluded from the present definition.

NNS are present in a wide range of foods and beverages, particularly products formulated to contain less or no added sugar. Their increasing use in the food supply has resulted in substantial exposure among children, including at young ages [4,5,6]. In the United States, national dietary data have documented consumption of low-calorie sweeteners in both children and adults [5]. More recent UK data show that low-calorie-sweetened products are commonly consumed by children and that their consumption may coexist with substantial intakes of free sugars and ultra-processed foods [6].

Exposure is also common in early childhood. In a recent study of 450 Chilean children assessed at 3 years of age, 76% consumed at least one NNS during the dietary assessment. Sucralose was the most frequently consumed sweetener (63%), followed by acesulfame K (35%), aspartame (32%), and steviol glycosides (27%); beverages were the main dietary source of NNS [7]. Notably, NNS intake was not associated with lower total sugar intake in this cohort [7]. These findings illustrate that exposure to NNS may begin well before adolescence and that the introduction of NNS into the diet does not necessarily correspond to a reduction in overall sugar consumption.

Early and repeated exposure is relevant to the present review because NNS preserve a strong sweet sensory signal while providing substantially less energy than sugars. Whether this difference between sensory sweetness and post-ingestive nutrient signaling influences appetite regulation or the development of food preferences is considered in the following sections.

## 4. Biological Mechanisms Linking Sweet Taste, Appetite and Food Preference

Appetite and food choice are shaped by the integration of orosensory signals generated before and during ingestion with metabolic and neural signals arising after nutrients enter the gastrointestinal tract. For sweet foods, oral sweetness and post-ingestive nutrient signaling represent related but partly separable sources of information that contribute to food reward and learned preference [12,13,14,15,16]. This distinction is particularly relevant to NNS, which preserve a strong sweet sensory signal while providing little or no energy.

### 4.1. Oral Sweet Taste Sensing

Sweet taste perception is initiated primarily through activation of the heterodimeric T1R2/T1R3 receptor expressed in taste receptor cells. This receptor responds to sugars as well as to several high-intensity sweeteners [12,23,24,25]. Activation of oral sweet taste pathways contributes to perceived sweetness and engages neural circuits involved in taste and reward [12,13,14,15,16].

Oral sweetness, however, does not fully determine the biological response to a sweet food or beverage. The rewarding value of a food is also influenced by signals generated after ingestion. Consequently, two substances perceived as similarly sweet may produce different metabolic and neural responses when their post-ingestive consequences differ [12,13,14,15,16].

### 4.2. Post-Ingestive Nutrient Sensing and Gut–Brain Signaling

After sugar ingestion, intestinal glucose generates signals that are partly independent of oral sweet taste. Sodium–glucose cotransporter 1 (SGLT1) contributes to intestinal glucose transport and sensing [26]. Pre-clinical studies have also identified rapid communication between enteroendocrine cells and vagal afferent neurons, providing a neural pathway through which luminal nutrient information can reach the brainstem [27]. Kaelberer et al. demonstrated such synaptic nutrient signaling using a mouse model, underscoring that this evidence is mechanistic rather than directly clinical.

Post-ingestive nutrient signals may also influence food reward and learned preference. In rodent models, intragastric or intestinal glucose can reinforce flavor preferences independently of oral sweetness, and vagal gut–brain signaling contributes to the development of sugar preference [16,28,29,30,31,32]. Tan et al., for example, identified gut–brain neural pathways that selectively support sugar preference in experimental animals. These findings demonstrate that sweetness and caloric value can provide distinct reinforcing signals, but they do not establish that the same mechanisms determine food choice in children.

### 4.3. Sweetness–Energy Coupling and Food Reward

With caloric sugars, sweet taste is usually followed by post-ingestive nutrient and energy signals. Repeated pairing of these sensory and metabolic consequences may contribute to learned associations between the taste of a food and its subsequent physiological effects [13,14,15,16,28,29,30,31,32]. NNS alter this relationship by providing sweetness with substantially less accompanying energy.

This sensory–metabolic dissociation has been proposed as a mechanism through which repeated NNS exposure might modify food reward, appetite regulation, or learned food preferences [17,18]. The hypothesis remains biologically plausible but is supported largely by experimental and animal data. NNS are also pharmacologically and metabolically heterogeneous, and there is no basis for assuming that all NNS generate the same post-ingestive signals or that they uniformly fail to engage gut–brain pathways.

Human evidence is limited and illustrates the importance of dietary context. In a short-term study in adults, Dalenberg et al. reported reduced insulin sensitivity and altered neural responses to sweet taste after sucralose was consumed together with carbohydrate, whereas sucralose consumed without carbohydrate did not produce the same effect [33]. Interpretation of this study has subsequently been debated, particularly with regard to the comparator and the inference that the observed effect could be attributed to low-calorie sweeteners themselves [34]. These findings therefore do not support a general conclusion that NNS disrupt neural reward signaling or increase appetite.

### 4.4. From Biological Plausibility to Clinical Relevance

Cephalic-phase insulin responses (CPIR) have also been proposed as a potential link between sweet taste exposure and subsequent metabolic or appetite responses. CPIR refers to an early insulin response elicited by sensory signals associated with food intake before the postabsorptive rise in circulating glucose. However, its occurrence, magnitude, and physiological relevance in humans are variable, and several aspects of its regulation and function remain unresolved [35]. Current evidence therefore does not justify assigning CPIR a major role in the effects of NNS on appetite.

**Figure 1:**
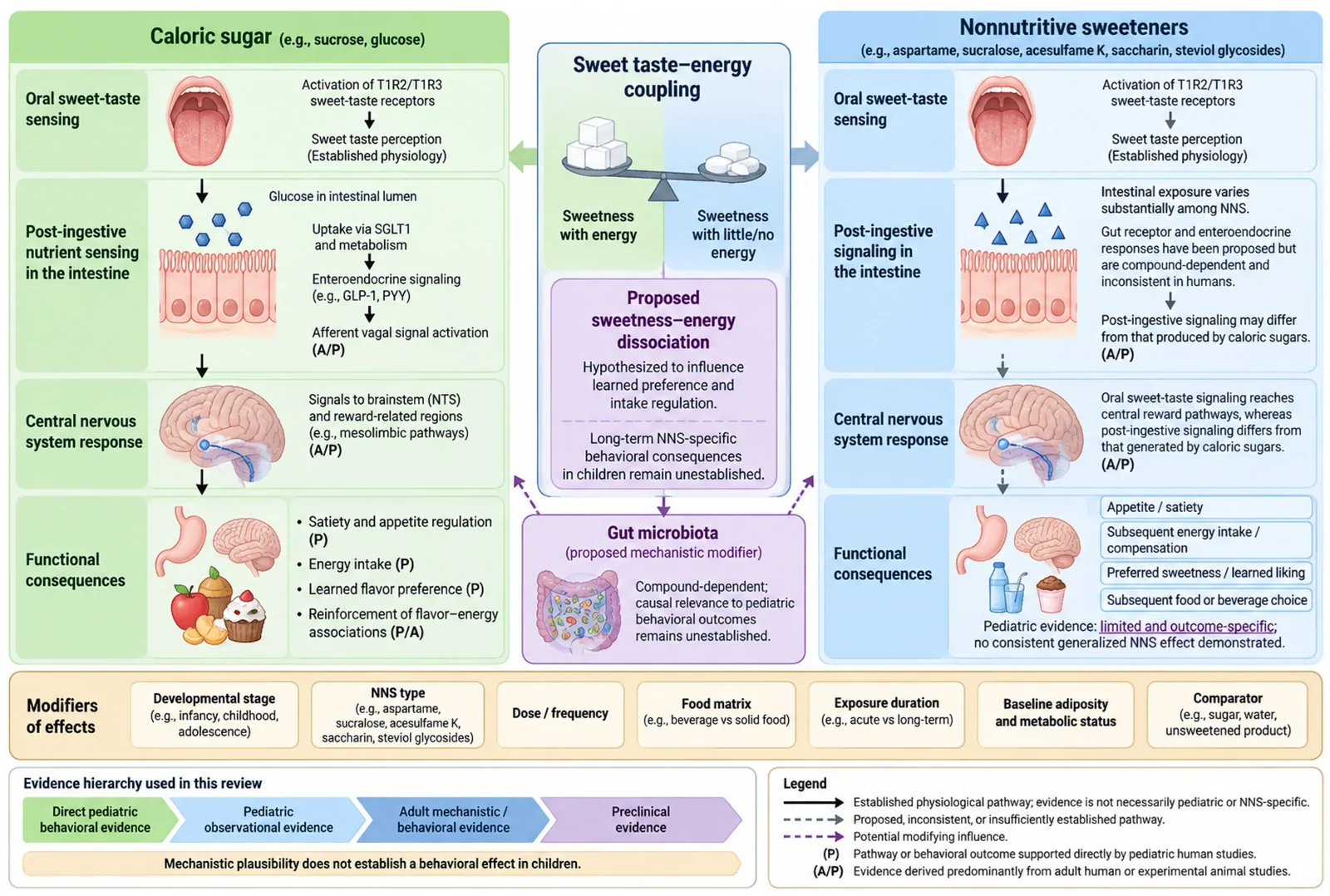
Conceptual model linking oral sweet-taste sensing, post-ingestive nutrient signaling, and potential behavioral effects of nonnutritive sweeteners.

The available mechanistic evidence supports biological plausibility rather than a demonstrated clinical effect. Much of the evidence linking post-ingestive nutrient sensing to learned preference derives from experimental animal models, while the limited human mechanistic studies have largely involved adults. Whether repeated NNS exposure alters appetite, satiety, energy intake, or food preference in children must therefore be established from pediatric clinical data rather than inferred from these mechanisms alone [12,16,27,33]. The principal pathways linking sweet taste, post-ingestive nutrient signaling, and the proposed effects of NNS on appetite and food preference are summarized in Figure 1.

## 5. Effects of NNS on Appetite, Satiety and Energy Intake

The hypothesis that NNS might alter appetite arises largely from the dissociation between sweet taste and energy delivery discussed in Section 4. Clinical studies have therefore examined subjective hunger and satiety, appetite-related hormonal responses, and subsequent energy intake. These outcomes should be distinguished from one another, and their interpretation depends strongly on the comparator: replacing sugar with NNS tests the effect of removing sugar-derived energy, whereas comparison with water or an unsweetened product is more informative about an effect attributable to NNS themselves [10,19].

Because these outcomes represent different constructs, the pediatric evidence is considered below according to the outcome directly measured rather than treating appetite, satiety, subsequent energy intake, and energy compensation as interchangeable indicators. Primary pediatric studies are emphasized, while evidence syntheses and adult studies are used mainly to provide context.

### 5.1. Effects on Appetite and Satiety

Direct pediatric evidence on subjective appetite and satiety is limited but includes both acute experimental studies and longer-term interventions. In two controlled experiments involving 20 healthy children aged 9–10 years in each experiment, Anderson et al. compared aspartame with either cyclamate in a carbohydrate-containing preload or sucrose in a sweetened beverage [36]. Hunger, fullness, and desire to eat were assessed repeatedly before a subsequent ad libitum lunch. Neither treatment comparison showed a significant effect on subjective appetite or lunch-time food intake.

Longer-term data are available from Frey et al., who randomized 126 apparently healthy children and adolescents to aspartame- or sucrose-containing diets for 13 weeks [37]. In the systematic reanalysis by Toews et al., self-reported increase in appetite (RR 0.84, 95% CI 0.22–3.29) and a decrease in appetite (RR 1.08, 95% CI 0.44–2.63) did not differ significantly between groups [11]. The wide confidence intervals, however, indicate substantial imprecision [11,37].

The strongest longer-term pediatric evidence comes from the DRINK trial [38]. In a prespecified substudy, 203 children aged 7–11 years were randomized to consume 250 mL/day of either a sugar-free beverage containing sucralose and acesulfame K or a matched sucrose-sweetened beverage for 18 months; 146 children completed the study. Satiety was assessed before and 1 and 15 min after beverage consumption at several time points during follow-up. No statistically significant differences were observed between groups: the adjusted odds ratio for a one-point greater increase in satiety in the sugar group was 0.77 (95% CI 0.46–1.29) at 1 min and 1.44 (95% CI 0.86–2.40) at 15 min. The sugar-sweetened beverage was, however, rated slightly higher for liking and wanting [38].

Taken together, these studies do not demonstrate a consistent effect of NNS on subjective appetite or satiety in children. However, this conclusion is based on a small and heterogeneous evidence base, with differences in age, sweetener type, comparator, exposure duration, and outcome measurement. Statistically nonsignificant differences should therefore not be interpreted as evidence of physiological equivalence between NNS and caloric sweeteners with respect to appetite regulation.

### 5.2. Effects on Energy Intake

Direct measurement of subsequent food intake provides information that cannot be inferred from subjective appetite ratings alone. Several pediatric preload studies illustrate that the response depends strongly on the caloric content of the comparator, timing of the test meal, and food matrix.

In two early experiments, Birch et al. examined children aged 2–5 years after preloads sweetened with sucrose, aspartame, aspartame plus maltodextrin, or water [39]. Aspartame-containing drinks reduced subsequent ad libitum food intake relative to water, particularly approximately 30 min after the preload, but the suppression was generally smaller than that observed after caloric sweet drinks. These findings suggest that both sensory and post-ingestive signals may contribute to short-term intake regulation and illustrate why an NNS-versus-water comparison addresses a different question from a NNS-versus-sugar comparison.

A larger study by Wilson examined 135 preschool children aged 18–66 months over a 12-week period in which lunches were served with plain milk, sucrose-sweetened chocolate milk, or aspartame-sweetened chocolate milk [40]. Beverage type did not significantly alter consumption of the other foods served at the meal. Because children drank more chocolate milk, however, total meal energy intake was higher when the sucrose-sweetened beverage was provided, whereas the aspartame-sweetened beverage increased beverage consumption without the additional energy supplied by sucrose.

In 15 normal-weight boys aged 9–14 years, van Engelen et al. compared isocaloric sucrose, high-fructose corn syrup, and glucose solutions with a noncaloric sucralose control consumed 60 min before an ad libitum meal [41]. Glucose, but not sucrose or high-fructose corn syrup, reduced subsequent meal intake relative to sucralose. Consequently, cumulative energy intake was higher after sucrose and high-fructose corn syrup than after the sucralose control. This small study demonstrates that even among caloric comparators, the metabolic substrate used may materially affect the apparent NNS effect.

Evidence from longer interventions is compatible with a caloric-displacement effect but remains difficult to interpret as an intrinsic effect of NNS on appetite. In the review by Toews et al., one randomized pediatric trial reported lower energy intake with a sucralose-containing intervention than with sucrose, but the estimate was imprecise and the certainty of evidence was low [11]. A separate controlled dietary study reported similar mean daily energy intake with aspartame and saccharin and higher intake during the sucrose condition.

Thus, reductions in energy intake observed when NNS replace caloric sweeteners should not be interpreted as evidence that NNS directly suppress appetite. They may instead reflect the removal of energy from the substituted product, with the magnitude of subsequent compensation varying according to age, comparator, timing, and food matrix.

### 5.3. Energy Compensation

A related question is whether the energy removed by replacing sugar with NNS is subsequently compensated for by greater food intake. Complete compensation would eliminate the energy deficit created by substitution, whereas partial compensation would preserve at least some of the reduction in total energy intake.

Pediatric experimental evidence suggests that caloric compensation is neither complete nor uniform. Rémy et al. studied 44 children aged 8–11 years using a within-subject conditioning paradigm in which distinctively flavored no-energy and 150 kcal sweet beverages were repeatedly paired with different energy densities [42]. After conditioning, subsequent meal intake was lower following the high-energy beverage than following the no-energy beverage, although compensation remained partial; after reversal of the flavor–energy association, caloric compensation was approximately 29%. The study also showed that learned liking could be influenced by previous associations between flavor and energy density, linking short-term energy regulation with the sweetness–energy learning mechanisms discussed in Section 4.

More recent work also highlights developmental heterogeneity. Reigh et al. studied 64 children aged 4–6 years in a five-condition crossover preload study that included regular apple juice, apple juice sweetened with an NNS, other apple forms, and a no-preload control [43]. The primary aim concerned food form rather than NNS specifically, but the protocol directly assessed subsequent ad libitum meal intake and energy compensation and identified sex-related differences in compensatory responses. Because the study was not designed primarily as an NNS-versus-sugar efficacy trial, its findings should be interpreted as supportive evidence on pediatric energy regulation rather than as evidence for a specific NNS effect.

Human intervention studies do not indicate consistent complete compensation. However, compensation should not be treated as a fixed characteristic of NNS exposure: available pediatric studies indicate that it may vary with developmental stage, sex, timing, energy density, and the sensory and nutritional characteristics of the comparator.

### 5.4. Interpretation of the Pediatric Evidence

The pediatric evidence on appetite, satiety, and spontaneous energy intake remains substantially smaller than the literature on body weight and adiposity. Importantly, the available studies do not address a single causal question. Acute preload experiments evaluate short-term appetite regulation; longer substitution trials address adaptation to repeated exposure; and dietary interventions often modify caloric intake together with sweetener exposure. Their estimates should therefore not be interpreted as interchangeable.

Across studies, there is no consistent evidence that NNS directly stimulate appetite or suppress satiety in children. At the same time, the evidence is insufficient to establish equivalence between NNS and caloric sweeteners, particularly for long-term appetite regulation. When lower total energy intake is observed with NNS, the finding is most consistently explained by replacement of sugar-derived energy combined with incomplete compensation, rather than by a specific anorexigenic action of the sweetener itself.

The evidence also indicates that the comparator is central to interpretation. NNS-versus-sugar studies primarily evaluate caloric substitution, whereas NNS-versus-water or no-preload comparisons are more informative regarding an effect attributable to the NNS-containing product itself. Sweetener type, food matrix, timing of exposure, age, and developmental stage further contribute to heterogeneity.

Primary pediatric studies directly addressing these outcomes are summarized in Table 2. Anthropometric and other indirect outcomes are considered separately in Section 7 and are not treated as surrogate measures of appetite or satiety.

## 6. Effects of NNS on Sweet Taste Preference and Food Choice

Humans show an early preference for sweet taste, although the strength of this preference varies substantially between individuals and changes across development [47]. Repeated dietary exposure may contribute to learned food preferences, raising the question of whether maintaining a high level of sweetness through NNS could influence subsequent sweet liking or food choice. This question is distinct from whether NNS reduce sugar or energy intake: replacing sugar may reduce its caloric contribution while leaving sensory exposure to sweetness largely unchanged.

Importantly, immediate liking or acceptance of an NNS-sweetened product, learned preference for a repeatedly experienced flavor, preferred sweetness intensity, and subsequent food or beverage choice represent related but distinct outcomes. Evidence for one should therefore not be interpreted as evidence for changes in the others.

### 6.1. Sweet Taste Exposure and Learned Preference

Direct pediatric evidence is limited but more informative when the specific behavioral outcome is considered. Jansen and Tenney examined taste-preference acquisition in 39 children with a mean age of approximately 5.5 years using nine conditioning trials in which caloric content and social modelling were experimentally varied [44]. Preference for the conditioned taste increased significantly only when caloric conditioning was combined with social modelling. The study therefore suggests that preference acquisition in young children may reflect both post-ingestive reinforcement and social context, rather than caloric content alone. However, it did not test whether repeated NNS exposure changes generalized preference for sweetness.

Longer-term data are provided by de Ruyter et al. In an 18-month double-blind trial, 203 children aged 7–11 years were randomized to receive a sugar-free beverage or a matched sugar-sweetened beverage [38]. Although satiety did not differ significantly between groups, children assigned to the sugar-sweetened beverage reported slightly greater liking and wanting of their assigned beverage (adjusted OR 1.63, 95% CI 1.05–2.54 for liking; OR 1.65, 95% CI 1.07–2.55 for wanting). These outcomes concern hedonic responses to the repeatedly consumed study beverage and should not be interpreted as evidence that NNS decrease, or sugar increases, the preferred intensity of sweetness more generally.

Immediate sensory acceptance has also been studied directly. In 89 children aged 5–12 years, de Ruyter et al. compared seven NNS-sweetened beverages with matched sugar-sweetened versions [45]. Only about one quarter of children reliably discriminated between the two types, and mean liking scores were identical for the NNS- and sugar-sweetened beverages (3.39 versus 3.39 on a 5-point scale; *p* = 0.9). This study demonstrates comparable immediate liking under controlled sensory conditions, but it does not address whether repeated exposure alters later sweet preference or food choice.

Rémy et al. provide more direct evidence regarding flavor–energy learning [42]. Forty-four children aged 8–11 years repeatedly consumed distinctly flavored sweet beverages presented in high-energy (150 kcal) and no-energy forms. After conditioning, liking for both beverages increased, but after a subsequent no-exposure period the flavor previously paired with the high-energy beverage was liked more than the flavor paired with the no-energy beverage. Following reversal of the flavor–energy association, the initially high-energy-paired flavor tended to retain its higher liking. These findings support the possibility of learned associations between sensory cues and post-ingestive energy in children, but the experimental manipulation concerned flavor–energy pairing rather than NNS exposure as such and does not demonstrate an increased preference for sweetness following NNS consumption.

More recently, Nakamura et al. evaluated child acceptance of school-lunch chocolate milk formulated with reduced sucrose and different nutritive and nonnutritive sweeteners [46]. In the child consumer experiment, lactose-hydrolyzed chocolate milks containing blends of monk fruit, stevia, and other sweetening strategies showed no difference in overall liking from the sucrose control. The study is relevant to product acceptance and demonstrates that NNS-containing formulations can achieve comparable immediate sensory acceptability under some formulation conditions. However, it does not address whether sustained exposure modifies subsequent preferred sweetness or later food choice.

Taken together, pediatric studies indicate that NNS-containing products can be accepted and liked by children and that flavor–energy associations can influence learned liking. They do not, however, establish that repeated NNS exposure increases the preferred level of sweetness or produces a persistent generalized preference for sweeter foods.

### 6.2. Adult Evidence as Behavioral Context

Adult intervention studies provide complementary context but should not be treated as direct evidence for children. In a randomized crossover study of 40 young adults, repeated exposure to sugar-sweetened and noncalorically sweetened soft drinks and yoghurt drinks did not significantly alter behavioral or neural measures of reward value after 10 exposures [48].

Similarly, the 6-month Sweet Tooth Trial found no between-group differences in sweet liking, perceived sweetness intensity, sweet food choice, energy intake, or body weight despite substantial differences in overall dietary sweet-taste exposure [49]. Importantly, this intervention manipulated sweetness from multiple dietary sources rather than NNS specifically. These findings argue against a simple relationship between the amount of dietary sweetness and subsequent sweet preference in adults, but they cannot establish the corresponding developmental response in children.

### 6.3. Food Choice and Dietary Patterns: Direct and Indirect Evidence

Evidence on actual subsequent food or beverage choice is considerably weaker than evidence on liking or product acceptance. The pediatric studies described above primarily assessed hedonic ratings, conditioning, discrimination, or immediate intake. They do not establish whether repeated exposure to NNS leads children to select sweeter foods or beverages in their habitual diet.

Observational studies provide information on the dietary context in which NNS are consumed, although they cannot determine whether NNS themselves alter food preference. In a nationally representative analysis of 5922 UK children aged 4–18 years, consumption of low- and no-calorie-sweetened products was not consistently associated with lower free-sugar intake over the 2008–2019 study period [6]. Children with higher consumption of these products also showed different overall dietary patterns, including lower water intake and less pronounced changes toward minimally processed foods and beverages [6].

Similarly, among 450 Chilean children assessed at 3 years of age, NNS intake was not significantly associated with lower total sugar intake [7]. These findings show that consumption of NNS-containing products does not necessarily correspond to a less sweet or lower-sugar overall dietary pattern. They should not, however, be interpreted as evidence that NNS cause greater preference for sweetness or alter subsequent food choice. Reverse causality and confounding by existing taste preferences, parental choices, product availability, food reformulation, and marketing remain substantial concerns.

### 6.4. Developmental Context and Early Exposure

Early childhood is a period during which beverage habits and food preferences are being established. Longitudinal data on sugar-sweetened beverages show that exposure during infancy predicts later consumption: in a US cohort of 1333 children, sugar-sweetened beverage intake during infancy was associated with a greater likelihood of daily consumption at 6 years of age [3]. This finding demonstrates tracking of beverage behavior but should not be interpreted as evidence that the same pattern occurs with NNS-containing products.

Overall, pediatric intervention studies provide evidence on immediate liking, beverage-specific wanting, and flavor–energy learning, but they do not directly establish whether sustained NNS exposure alters the preferred intensity of sweetness or subsequent habitual food choice. The current evidence should therefore be interpreted as insufficient to demonstrate either a persistent increase or a persistent decrease in sweet preference following NNS exposure. Long-term pediatric interventions specifically designed to measure changes in preferred sweetness and subsequent food selection remain a major research need.

## 7. Body Weight and Adiposity as Contextual Outcomes

Evidence relating NNS consumption to body weight in children differs according to study design. Randomized trials predominantly evaluate the replacement of sugar-sweetened beverages with NNS-containing alternatives, whereas observational studies assess habitual NNS consumption within existing dietary and weight-control behaviors. These designs therefore address different questions and are differently susceptible to confounding and reverse causality [9].

Anthropometric outcomes are considered here as clinically relevant contextual evidence rather than as surrogate measures of appetite, sweet taste preference, or food choice. Changes in BMI or adiposity cannot establish that NNS have altered eating-related behavior.

### 7.1. Randomized Trials: Replacing Sugar with NNS

The most recent pediatric systematic review and meta-analysis by Espinosa et al. included four randomized controlled trials comprising 1372 participants and eight prospective cohort studies including 35,340 participants [9]. In the randomized trials, assignment to NNS-containing beverages rather than sugar-sweetened beverages was associated with slightly less BMI gain (mean difference −0.114 kg/m^2^; 95% CI −0.207 to −0.021). However, between-study heterogeneity was substantial (I^2^ = 87%), indicating that the pooled estimate summarizes trials with markedly variable effects and should not be interpreted as a uniform treatment effect across pediatric populations [9].

Stratified analyses showed less BMI gain among adolescents and participants with obesity at baseline, as well as in longer trials and trials using mixtures of NNS [9]. These subgroup findings may help explain part of the observed heterogeneity, but they should be considered exploratory because they are based on a small number of trials.

The comparator is central to interpretation. All randomized trials contributing to the pooled estimate compared NNS-containing beverages with sugar-sweetened beverages; no eligible pediatric trial compared NNS-containing beverages directly with water [9]. Thus, the observed effect is best interpreted as a consequence of replacing sugar-derived energy rather than evidence that NNS themselves promote weight loss. Earlier meta-analyses similarly found that estimates of body-weight effects depend strongly on the comparator and the clinical context [10,50].

### 7.2. Prospective Observational Studies

In the same meta-analysis, prospective cohort studies did not show a statistically significant association between NNS-containing beverage consumption and subsequent BMI change (0.05 kg/m^2^ per additional daily 355 mL serving; 95% CI −0.03 to 0.13). Heterogeneity was again substantial (I^2^ = 75%), and the overall quality of evidence was rated as low to moderate [9]. The absence of a statistically significant pooled association should therefore be interpreted as insufficient evidence for a consistent prospective relationship, rather than as evidence that no relationship exists.

Some earlier observational studies reported positive associations between NNS-containing beverage intake and higher BMI or adiposity [9,11]. These findings are particularly vulnerable to reverse causality because NNS use may increase after weight gain or in response to attempts to reduce sugar intake. Residual confounding by baseline adiposity, dietary pattern, physical activity, and weight-control behavior further limits causal interpretation.

### 7.3. Body Composition and Adiposity

BMI is practical for population studies but does not directly distinguish fat mass from fat-free mass [49]. This is particularly relevant during childhood and adolescence, when growth and pubertal development are accompanied by substantial changes in body composition.

Chien et al. examined longitudinal associations between individual sweetener intake and body composition in 1893 children aged 6–15 years [51]. Associations differed by sweetener, sex, and nutritional status, with some exposures associated with lower fat mass or greater fat-free mass. However, several estimates included the null, the study evaluated multiple exposures and outcomes, and residual confounding cannot be excluded. In addition, sorbitol, which was included among the studied compounds, is a polyol rather than an NNS according to the WHO terminology used in this review [21]. These findings are therefore more informative about possible compound- and developmental-stage-specific heterogeneity than about an overall effect of NNS on body composition.

### 7.4. Cross-Sectional Evidence

Cross-sectional studies have also reported positive associations between sugar-free beverage consumption and overweight or adiposity. For example, Zhang et al. found higher odds of overweight/obesity and abdominal obesity with greater sugar-free beverage intake among 3227 Chinese children and adolescents aged 9–17 years [52]. However, because exposure and adiposity were assessed at the same time, these data cannot establish temporal direction and are highly susceptible to reverse causality.

Across study designs, the available evidence does not support a uniform effect of NNS on pediatric adiposity. Randomized trials suggest slightly less BMI gain when NNS-containing beverages replace sugar-sweetened beverages, although the magnitude of this effect is heterogeneous. Prospective observational studies do not demonstrate a consistent association with BMI change, whereas positive cross-sectional associations are particularly susceptible to reverse causality and residual confounding.

These apparently discordant findings are therefore compatible with differences in comparator, study design, population, and exposure context rather than requiring opposing biological effects of NNS. Most importantly for the present review, anthropometric outcomes cannot determine whether NNS alter appetite, preferred sweetness, or subsequent food choice.

## 8. Developmental Context and Early-Life Exposure

NNS exposure may occur during pregnancy, lactation, and throughout childhood, but these periods represent biologically and behaviorally distinct exposure contexts. Developmental stage is particularly relevant when evaluating appetite and food preference because preschool children, school-age children, and adolescents differ in energy requirements, sweet taste preference, dietary autonomy, pubertal physiology, and the social context of food choice. Early-life exposure and developmental modifiers should therefore be considered as potential sources of heterogeneity rather than treated as a single pediatric exposure category.

### 8.1. Prenatal and Lactational Exposure: Contextual Evidence

Prenatal exposure to NNS has been examined primarily in relation to offspring growth and adiposity rather than later appetite or food preference. Prospective cohorts, including the Canadian CHILD study and Project Viva, have reported associations between greater maternal intake of artificially sweetened beverages and higher offspring BMI or adiposity measures [53,54]. However, these observational findings are susceptible to residual confounding by maternal adiposity, diabetes, smoking, dietary pattern, and weight-control behavior.

A 2025 systematic review and meta-analysis of 17 studies found no consistent association between maternal low-calorie sweetener consumption during pregnancy and offspring BMI z-score, while estimates for overweight and several secondary outcomes remained imprecise and the quality of evidence was low to very low [55]. Evidence during lactation is even more limited; in the Infant Feeding Practices Study II, estimated infant exposure through maternal diet-beverage consumption was not associated with BMI-for-age z-score or overweight at 3 months [56].

Importantly for the present review, these studies do not directly assess whether prenatal or lactational NNS exposure influences later appetite regulation, preferred sweetness, or food choice. Maternal exposure should therefore be regarded as developmental context rather than direct evidence for the behavioral outcomes considered in this review.

### 8.2. Developmental Programming: Hypotheses Rather than Demonstrated Behavioral Effects

Developmental programming has been proposed as a potential mechanism linking early NNS exposure with later metabolic or behavioral outcomes. However, direct human evidence that prenatal NNS exposure alters subsequent sweet preference or eating behavior is lacking [4,20].

Differences in infant gut microbiome composition and metabolites have also been reported in association with maternal artificially sweetened beverage intake, including in a subset of the CHILD cohort [57]. These observations remain hypothesis-generating: observational mediation analyses cannot establish a causal pathway from maternal NNS exposure through the microbiome to later appetite, food preference, or adiposity.

### 8.3. Developmental Stage: Preschool Childhood, School Age, and Adolescence

Children should not be treated as a single developmental population. The relevance and interpretation of NNS exposure differ across preschool childhood, school age, and adolescence.

During preschool childhood, food preferences are still being actively shaped by repeated exposure, parental feeding practices, and social learning. NNS exposure is already common at this age, as shown in Chilean preschool cohorts, but most available studies characterize prevalence rather than subsequent changes in preferred sweetness or food choice [58,59]. Experimental studies in young children, including preload and conditioning paradigms, demonstrate that short-term intake and learned liking can be modified by energy content, timing, and social context, but they do not establish a long-term NNS-specific effect on sweet preference.

During school age, children have greater dietary autonomy while still remaining substantially influenced by family and school food environments. Several of the most informative direct pediatric studies fall within this age range, including the 18-month DRINK trial and conditioning experiments examining satiety, beverage liking, energy compensation, and flavor–energy learning. These studies provide evidence for specific proximal behavioral responses but do not demonstrate that sustained NNS exposure alters generalized preferred sweetness.

Adolescence introduces additional heterogeneity through puberty, increasing dietary autonomy, greater exposure to commercially available beverages, and more frequent weight-control behavior. Randomized substitution trials suggest that effects on BMI gain may differ according to age and baseline adiposity [9], but anthropometric responses should not be interpreted as evidence that appetite or food preference has changed.

### 8.4. Sex, Puberty, and Baseline Adiposity

Sex, pubertal stage, and baseline adiposity may modify observed associations. In the longitudinal study by Chien et al., associations between individual sweeteners and changes in fat mass and fat-free mass differed according to sex and nutritional status [60]. These subgroup findings do not establish true biological interaction, but they support treating developmental and anthropometric characteristics as potential effect modifiers rather than merely adjustment variables.

More recent experimental evidence also suggests that short-term energy compensation may differ by sex. In the crossover study by Reigh et al. in children aged 4–6 years, compensatory responses following different apple preloads varied between boys and girls. Because the study was designed primarily around food form rather than NNS-specific effects, these findings should be considered exploratory, but they further illustrate the possibility of developmental and sex-related heterogeneity.

Baseline adiposity is also relevant because it may act both as a potential effect modifier and as a source of reverse causality. Children with overweight or obesity may increase NNS consumption after weight gain or as part of attempts to reduce sugar and energy intake. Conversely, some randomized substitution trials suggest larger differences in BMI gain among adolescents and participants with obesity [9]. These findings should not be interpreted as evidence that NNS exert different effects on appetite or food preference without direct behavioral measurements.

Overall, developmental stage should be treated as an integral component of study interpretation rather than as a demographic detail. Preschool children, school-age children, and adolescents differ in exposure patterns, physiological development, dietary autonomy, and the mechanisms through which food preferences are learned. However, current evidence remains insufficient to determine whether the long-term behavioral effects of NNS differ systematically across these developmental stages.

## 9. Sources of Heterogeneity and Methodological Limitations

Apparent inconsistencies across pediatric studies of NNS reflect substantial variation in exposure definition, comparator, study design, participant characteristics, outcome selection, and duration of follow-up. Acute experimental studies, randomized substitution trials, prospective cohorts, and cross-sectional analyses address different causal questions and are subject to different sources of bias [4,9,10,11]. Their findings should therefore not be interpreted as directly interchangeable or combined into a single overall concept of an “NNS effect.”

### 9.1. Heterogeneity of NNS Exposure

Accurate characterization of NNS exposure remains difficult. Some studies identify individual sweeteners, whereas others combine several compounds or use broad categories such as “diet beverages”, “sugar-free beverages”, or “artificially sweetened beverages” as proxies for NNS intake. Food-frequency questionnaires and self-reported product consumption may provide limited information on the specific compound, dose, or combination of sweeteners consumed. In the systematic review by Toews et al., inadequate reporting of the sweetener used and substantial variation in reported doses and outcome measures limited study inclusion and prevented several dose-specific comparisons [11].

NNS should also not be considered a uniform biological exposure. Individual compounds differ in chemical structure, sweetness intensity, absorption, metabolism, and gastrointestinal exposure [4,22]. Studies additionally vary in dose, frequency of consumption, food matrix, and whether individual sweeteners or mixtures are used. Consequently, findings obtained with one compound or formulation cannot automatically be generalized to NNS as a class. Dose should also be interpreted in relation to body size and developmental stage. Because exposure is commonly expressed per kilogram of body weight, younger children may receive a higher weight-adjusted dose from the same product than older children; this pattern has been reported for several individual NNS in Chilean children [61].

Changes in food formulation introduce an additional source of exposure misclassification. Studies conducted before and after Chilean food-labeling legislation documented marked changes in both the prevalence and type of NNS consumed following product reformulation [58,59,61]. A stable product category may therefore conceal substantial changes in the specific sweeteners consumed over time.

### 9.2. Comparator, Food Matrix, and Study Design

The comparator determines the causal question addressed by an intervention study. Trials comparing NNS with sugar primarily evaluate the consequences of caloric substitution, whereas comparisons with water or unsweetened products are more informative about effects attributable to the NNS-containing product itself. The absence of an appropriate unsweetened comparator therefore limits inference about an intrinsic NNS effect [9,10].

The food matrix adds another layer of heterogeneity. An NNS consumed in a beverage, dairy product, or mixed meal may differ in gastric emptying, concurrent nutrient delivery, sensory characteristics, and the timing of post-ingestive signals. Results from an NNS-sweetened preload therefore cannot necessarily be extrapolated to habitual consumption of NNS across multiple foods and beverages.

Study design further determines interpretation. Randomized trials assign a specific substitution and provide greater control over exposure, whereas observational studies capture NNS consumption within pre-existing dietary habits, body-weight trajectories, and attempts to modify diet. These designs should not be expected to yield identical estimates even when nominally similar outcomes are assessed [9,10,11].

### 9.3. Reverse Causality and Residual Confounding

Reverse causality is a major limitation of observational studies of NNS and adiposity. Children with overweight or obesity may increase consumption of NNS-containing products following weight gain, parental concern, or attempts to reduce sugar and energy intake. An association between greater NNS consumption and higher BMI may therefore reflect an effect of body weight on beverage choice rather than an effect of NNS on body weight [9,11].

Residual confounding presents a related challenge because NNS consumption may cluster with baseline adiposity, dieting behavior, physical activity, socioeconomic factors, and broader dietary patterns [6]. Similar concerns apply to prenatal studies, in which maternal NNS intake may correlate with pre-pregnancy adiposity, diabetes, weight-control behavior, and overall diet [53,54,55]. Observational associations in these settings should therefore not be interpreted as causal without appropriate consideration of these factors.

### 9.4. Non-Equivalent Outcome Definitions

A major source of apparent inconsistency is the use of non-equivalent outcomes. Subjective hunger, satiety, ad libitum energy intake, energy compensation, product liking, preferred sweetness intensity, habitual food choice, BMI, BMI z-score, fat mass, and metabolic responses represent different constructs. A change in one cannot be assumed to imply a corresponding change in another.

For example, unchanged subjective appetite ratings do not exclude differences in subsequent energy intake; comparable immediate liking of an NNS-sweetened product does not establish that repeated exposure leaves preferred sweetness unchanged; and a difference in BMI does not demonstrate a change in appetite or food preference.

This distinction is particularly important when integrating evidence across reviews. Anthropometric outcomes should not be used as surrogate evidence for behavioral effects, and immediate sensory acceptance should not be treated as equivalent to learned or habitual food preference.

Similarly, statistically nonsignificant findings should not automatically be interpreted as evidence of equivalence or absence of effect, particularly in small studies with imprecise estimates.

### 9.5. Duration of Exposure and Mismatch Between Study Questions

Duration of exposure creates an additional mismatch between the questions that can be answered experimentally and those of greatest developmental interest. Acute preload studies can characterize short-term appetite, satiety, subsequent food intake, and energy compensation under controlled conditions, but they cannot determine whether repeated exposure changes preferred sweetness or habitual food choice over months or years. Conversely, studies with the longest follow-up are predominantly observational and therefore provide less control over exposure, comparator, and confounding.

This limitation is particularly relevant to the central question of whether sustained NNS exposure alters learned food preference. Existing pediatric studies provide information on immediate liking, short-term intake regulation, flavor–energy conditioning, and beverage-specific responses, but direct long-term evidence on changes in preferred sweetness intensity or habitual food selection remains sparse.

Systematic reviews reflect this limitation. Toews et al. noted that many available studies were small, of short duration, and affected by methodological or reporting limitations [11], while Espinosa et al. synthesized randomized trials and prospective cohorts representing different exposure contexts and causal questions [9].

### 9.6. Integrating Different Levels of Evidence

The different evidence streams considered in this review therefore serve different purposes. Pediatric randomized and controlled studies provide the most direct evidence for short-term behavioral effects; pediatric observational studies characterize habitual exposure and longer-term associations but remain susceptible to confounding and reverse causality. Adult studies provide complementary behavioral and mechanistic context, while animal experiments primarily establish biological plausibility. Findings from these evidence levels should inform one another but should not be treated as equivalent confirmation of an effect in children.

Likewise, multiple systematic reviews may include overlapping primary studies. Concordant conclusions from such reviews should not be interpreted as independent replication when they are based on the same underlying trials.

The uncertainty surrounding pediatric NNS effects therefore does not arise from a single contradictory finding. It reflects heterogeneity in the sweetener and dose studied, food matrix, comparator, developmental stage, exposure duration, study design, and outcome definition, together with imprecision, reverse causality, and residual confounding. Consequently, apparently discordant results may often represent different questions rather than genuinely conflicting answers.

The clearest interpretation emerges when evidence is organized by the direct outcome measured and by the comparator used, while keeping anthropometric, observational, adult, and preclinical evidence in their appropriate contextual roles.

## 10. Clinical Interpretation and Practical Implications

Current pediatric evidence does not support an intrinsic role for NNS in appetite suppression, modification of sweet preference, or obesity treatment. Their potential clinical relevance depends primarily on what they replace. When NNS-containing products replace sugar-sweetened alternatives, they may reduce sugar and energy intake and may be associated with slightly less BMI gain. These substitution effects should be distinguished from effects attributable to NNS themselves. Evidence comparing NNS with water or unsweetened products remains substantially more limited [9,10].

Likewise, the available pediatric studies do not demonstrate that NNS consistently increase appetite, increase preferred sweetness, or promote less healthy food choice. However, the evidence is insufficient to exclude long-term behavioral effects during development.

### 10.1. Weight Control and Sugar Substitution

The 2023 WHO guideline recommends against the use of non-sugar sweeteners as a means of achieving long-term weight control or reducing the risk of noncommunicable diseases [21]. The recommendation applies to adults and children, except individuals with pre-existing diabetes, and is conditional because of limitations in the available evidence [21]. This public-health recommendation is distinct from toxicological assessments of individual sweeteners and does not replace acceptable daily intake values established by regulatory authorities.

Accordingly, NNS-containing products should not be presented as a treatment for obesity or as an independent strategy for appetite control. However, this does not imply that substitution is clinically irrelevant. In children and adolescents with a high habitual intake of sugar-sweetened beverages, replacing those beverages with NNS-containing alternatives may reduce free-sugar and energy intake. Pediatric randomized trials also suggest slightly less BMI gain when NNS-containing beverages replace sugar-sweetened beverages, although the magnitude of this effect is heterogeneous [9].

This should be interpreted as a substitution effect rather than evidence of an intrinsic weight-reducing action of NNS. The AAP similarly concluded that replacing caloric sweeteners with NNS may provide modest short-term weight benefit, while NNS use alone is unlikely to produce substantial weight loss [20].

### 10.2. Water and Unsweetened Beverages Remain the Preferred Replacements

When the objective is to reduce free-sugar intake, water should remain the preferred replacement for sugar-sweetened beverages. ESPGHAN recommends replacing free sugars in liquid form with water or unsweetened milk drinks [2], and the Polish Society for Pediatric Gastroenterology, Hepatology and Nutrition similarly emphasizes reducing free-sugar intake and limiting sugar-sweetened beverages [62]. NNS-containing beverages may provide a lower-energy alternative to sugar-sweetened beverages, but this does not make them equivalent to water or unsweetened beverages as the preferred long-term dietary option.

For a child with a high habitual intake of sugar-sweetened beverages who is unable to transition directly to water or unsweetened alternatives, replacement with an NNS-containing beverage may represent a pragmatic step for reducing sugar and energy intake. This is an interpretation of the substitution evidence rather than a recommendation directly evaluated in clinical trials or specifically endorsed as a stepwise strategy in current guidelines. Where feasible, the longer-term objective remains greater reliance on water and less-sweet beverages [2,21,62].

### 10.3. Young Children: Limited Evidence Requires Cautious Interpretation

Evidence on NNS use in infants and very young children is particularly sparse. The AAP concluded that no specific advice could be provided for children younger than 2 years because of the absence of adequate data [20]. More broadly, direct pediatric studies examining the long-term effects of NNS on sweet liking, food choice, and spontaneous energy intake remain limited [4,20].

The available evidence therefore does not provide a basis for recommending routine use of NNS-containing products in young children as a strategy to establish healthy eating or beverage habits. This reflects insufficient evidence and the developmental importance of early dietary learning, rather than evidence that approved NNS are harmful.

Product descriptors such as “zero”, “light”, “sugar-free”, or “no added sugar” should also not be interpreted as indicators of overall nutritional quality. Parents may have difficulty identifying NNS in products marketed to young children; in a study of 1603 US parents of children aged 1–5 years, more than half were unable to correctly identify beverages containing NNS from front-of-package information [8].

### 10.4. Counseling Should Focus on the Overall Dietary Pattern

NNS consumption occurs within a broader dietary pattern. Observational data from UK children show that consumption of low- and no-calorie-sweetened products was not consistently associated with lower free-sugar intake and occurred alongside differences in overall food and beverage choices [6]. An NNS-containing product should therefore not be considered nutritionally preferable solely because it contains less or no added sugar.

Dietary counseling should focus primarily on the overall pattern: reducing sugar-sweetened beverages, using water as the default beverage, favoring whole fruit over sweet drinks, and selecting unsweetened foods and dairy products where appropriate [2,62]. The specific role of an NNS-containing product is best judged according to what it replaces, how frequently it is consumed, and how it fits within the child’s habitual diet.

Communication with families should avoid overstating either benefit or harm. Regulatory authorization of an individual NNS addresses safety under specified conditions of use; it does not establish long-term metabolic or behavioral benefit. Conversely, uncertainty regarding long-term pediatric outcomes should not be interpreted as evidence that approved NNS are inherently harmful.

## 11. Future Research Priorities

Pediatric research on NNS has focused predominantly on exposure prevalence, anthropometric outcomes, and metabolic associations, whereas appetite, spontaneous energy intake, preferred sweetness, learned preference, and actual food choice have been studied much less extensively [4,9,11,20]. The principal research priority is therefore not simply to generate more pediatric NNS studies, but to design studies that directly address the behavioral questions for which evidence remains insufficient.

### 11.1. Direct Behavioral Outcomes Should Be Measured Separately

Future pediatric studies should distinguish subjective appetite, satiety, subsequent energy intake, energy compensation, immediate product liking, preferred sweetness intensity, learned preference, and habitual food choice rather than treating them as interchangeable indicators of eating behavior. Relevant endpoints should therefore include validated appetite ratings, ad libitum energy intake, preferred sweetness concentration, repeated measures of sweet liking, and subsequent beverage or food selection.

A particularly important unanswered question is whether sustained exposure to NNS alters the preferred level of sweetness over time. Existing pediatric studies provide information on immediate acceptance, beverage-specific liking and wanting, short-term intake regulation, and flavor–energy learning, but they do not directly establish whether repeated NNS exposure changes generalized preferred sweetness or subsequent habitual food choice.

Longitudinal intervention studies should therefore assess behavioral outcomes both during exposure and after the intervention has ended, allowing transient sensory responses to be distinguished from persistent changes in preference.

### 11.2. NNS Exposure and Comparator Should Be Defined Precisely

Future studies should identify the specific sweetener or sweetener mixture used and report dose, frequency, duration of exposure, and food matrix. NNS should not be treated as a biologically uniform exposure, because individual compounds differ in absorption, metabolism, gastrointestinal exposure, and sweetness characteristics [22].

Comparator selection should be defined according to the causal question. NNS-versus-sugar comparisons primarily evaluate caloric substitution, whereas NNS-versus-water or unsweetened comparators are necessary to examine effects attributable to the NNS-containing product beyond the removal of sugar-derived energy. Whenever feasible, pediatric intervention studies should include both caloric and unsweetened comparator conditions [9,10].

Studies should also distinguish direct exposure to NNS from broader manipulation of dietary sweetness. Reducing or increasing overall sweet-taste exposure through multiple food sources is not equivalent to changing exposure to a specific NNS.

### 11.3. Developmental Stage and Duration of Exposure Should Be Incorporated Prospectively

Children should not be treated as a single developmental population. Preschool childhood, school age, and adolescence differ in growth, energy requirements, pubertal physiology, dietary autonomy, and the social processes through which food preferences are learned. Future studies should therefore prospectively characterize age, sex, pubertal stage, and baseline adiposity and examine these variables as potential effect modifiers rather than only as covariates [9,60].

Study duration should also match the outcome of interest. Acute experiments remain useful for appetite, satiety, postprandial responses, and short-term energy compensation, but they cannot determine whether repeated NNS exposure alters learned preference or habitual food choice. Longer randomized interventions with post-intervention follow-up are needed to determine whether any behavioral effects persist after active exposure ends.

Studies of early-life exposure should distinguish maternal consumption during pregnancy, exposure during lactation, and direct consumption by the child because these represent biologically and behaviorally distinct exposure periods. Where such cohorts are available, later behavioral outcomes—including preferred sweetness and food choice -would be more informative for the questions addressed in this review than anthropometric outcomes alone.

### 11.4. Mechanistic Hypotheses Should Be Tested Against Behavioral Outcomes

Mechanistic and behavioral outcomes are rarely measured within the same pediatric study. Future experiments should, where feasible, combine measures of sweet taste perception and post-ingestive nutrient signaling with direct behavioral endpoints such as appetite, ad libitum intake, preferred sweetness, and subsequent food choice. This would allow proposed mechanisms to be tested against clinically meaningful behavior rather than inferred from adult or preclinical findings alone.

Such designs would be particularly informative for the proposed dissociation between oral sweetness and post-ingestive energy signaling. Experimental evidence supports a role for these signals in food reward and learned preference [12,16,27], but whether repeated sweetness–energy dissociation produces persistent behavioral effects during childhood remains unresolved.

Microbiome and other mechanistic biomarkers may provide complementary information but should remain exploratory unless they can be linked prospectively to clearly defined behavioral outcomes.

### 11.5. Evidence Synthesis Should Preserve Study-Level Distinctions

Future evidence syntheses should distinguish direct pediatric behavioral studies from anthropometric or metabolic studies and from mechanistic adult or animal evidence. Acute experiments, long-term randomized substitution trials, and observational cohorts should not be treated as addressing the same causal question. Likewise, multiple reviews containing overlapping primary trials should not be interpreted as independent replication of the underlying evidence.

## 12. Conclusions

Current pediatric evidence does not demonstrate a consistent direct effect of nonnutritive sweeteners on subjective appetite or satiety. Short-term studies of subsequent energy intake and energy compensation show context-dependent responses, whereas reductions in total energy intake are most consistently observed when NNS replace caloric sugars. These findings support a caloric-substitution effect rather than an intrinsic appetite-suppressing action of NNS.

Evidence concerning sweet taste preference is more limited. Pediatric studies show that NNS-containing products can be accepted and liked, and experimental conditioning studies demonstrate that flavor–energy associations can influence learned liking. However, available studies do not establish that sustained NNS exposure alters generalized preferred sweetness intensity. Likewise, direct evidence that repeated NNS exposure changes subsequent habitual food or beverage choice in children remains sparse.

Interpretation of the available literature depends strongly on the specific sweetener, dose, food matrix, comparator, duration of exposure, developmental stage, and outcome assessed. NNS-versus-sugar trials primarily evaluate substitution of sugar-derived energy, whereas comparisons with water or unsweetened products are more informative about effects attributable to NNS-containing products themselves. Anthropometric outcomes, observational dietary patterns, and mechanistic findings from adults or animal models provide relevant context but should not be treated as equivalent to direct pediatric behavioral evidence.

For clinical practice, reducing free-sugar intake remains the primary objective, with water and unsweetened foods and beverages preferred when feasible [2,21,62]. NNS-containing products may reduce sugar and energy intake when they replace sugar-sweetened alternatives, but current evidence does not support their routine use specifically to suppress appetite, prevent obesity, or modify food preferences. Importantly, the absence of a demonstrated behavioral effect should not be interpreted as evidence that long-term developmental effects are absent. Well-designed pediatric studies directly assessing preferred sweetness and subsequent food choice over prolonged exposure remain a major research priority.

## Figures and Tables

**Table 1:** Selected nonnutritive sweeteners: major absorption and metabolic characteristics relevant to interpretation of pediatric appetite and food-preference studies.

Sweetener	E Number	Approximate Sweetness vs. Sucrose *	Major Absorption and Metabolic Characteristics	Relevance for Interpretation of Behavioral and Mechanistic Studies
Aspartame	E951	~200×	Extensively hydrolyzed in the gastrointestinal tract before systemic absorption to phenylalanine, aspartic acid, and methanol; systemic exposure is therefore primarily to these breakdown products rather than to intact aspartame.	Its disposition differs fundamentally from poorly absorbed NNS. Experimental findings requiring substantial distal intestinal exposure to intact sweetener should therefore not be generalized directly to aspartame.
Sucralose	E955	~600×	Absorption in humans is low (generally ≤20%); most ingested sucralose remains unabsorbed and is excreted largely unchanged, predominantly in feces, while the absorbed fraction is excreted mainly in urine.	Compared with aspartame, a larger proportion of intact sweetener reaches the distal gastrointestinal tract, which may be relevant when considering gut-mediated signaling or microbiome hypotheses.
Acesulfame potassium (Ace-K)	E950	~200×	Almost completely absorbed, not detectably metabolized, and rapidly excreted primarily unchanged in urine.	Systemic exposure is to the parent compound. Ace-K is frequently used in sweetener mixtures, which may prevent attribution of observed effects to Ace-K alone.
Saccharin	E954	~300×	Well absorbed in humans, essentially not metabolized, and primarily excreted unchanged in urine; reported elimination half-life is approximately 4 h.	Its pharmacokinetic profile differs from both poorly absorbed compounds and sweeteners requiring microbial transformation. Systemic exposure to the parent compound should be considered when comparing mechanistic studies.
Steviol glycosides	E960	~200–300×	Not substantially hydrolyzed by digestive enzymes in the upper gastrointestinal tract. They reach the colon, where gut microbiota convert them to steviol; steviol is subsequently absorbed, glucuronidated, and excreted mainly as steviol glucuronide.	Microbiota-dependent conversion and substantial colonic exposure distinguish steviol glycosides from rapidly absorbed or pre-absorptively hydrolyzed NNS and may be relevant to gut-mediated mechanisms.

* Relative sweetness values are approximate and vary with the specific compound, concentration, food matrix, and formulation. The table summarizes major pharmacokinetic characteristics relevant to interpretation of mechanistic and behavioral studies and is not intended as a comprehensive toxicological or safety assessment.

**Table 2:** Pediatric studies directly assessing appetite, satiety, energy intake, sweet taste liking, and food-related behavioral outcomes following exposure to nonnutritive sweeteners.

Study	Population and Design	NNS /Comparator	Exposure	Direct Outcome	Main Finding	Interpretation/Key Limitation
Birch et al., 1989 [39]	Two controlled preload experiments in children aged 2–5 y	Aspartame vs. sucrose, aspartame + maltodextrin, or water	Acute; preload followed by ad libitum food intake at different intervals	Subsequent food intake; energy compensation	Subsequent intake varied according to preload composition and timing. Aspartame-containing drinks suppressed intake relative to water under some conditions, but generally less than caloric sweet drinks.	Demonstrates that comparator and timing materially influence the apparent effect. Small samples and acute exposure limit inference about habitual NNS use.
Anderson et al., 1989 [36]	Two experiments, each *n* = 20 healthy children aged 9–10 y	Aspartame vs. cyclamate with carbohydrate; aspartame vs. sucrose	Acute; treatment 90 min before ad libitum lunch	Hunger, fullness, desire to eat; lunch-time food intake	Treatments did not significantly affect subjective appetite or subsequent food intake.	Direct assessment of appetite and intake, but small samples and single-exposure design.
Wilson, 2000 [40]	135 children aged 18–66 months; repeated-meal study	Plain milk vs. sucrose-sweetened chocolate milk vs. aspartame-sweetened chocolate milk	Six presentations of each menu/beverage condition over 12 weeks	Meal food intake; beverage intake; total meal energy intake	Beverage type did not significantly alter intake of other foods. Children consumed more chocolate milk than plain milk; total meal energy intake was higher with sucrose-sweetened chocolate milk.	Suggests incomplete compensation for beverage energy. Milk matrix, flavor and palatability prevent attribution to NNS alone.
Jansen and Tenney, 2001 [44]	39 completers; mean age 67 months; 2 × 2 conditioning experiment	Energy-containing vs. artificially sweetened low-energy product, with or without social modelling	Nine conditioning trials	Acquisition of taste preference	Preference increased significantly only when caloric conditioning was combined with social modelling.	Supports a role for both post-ingestive and social learning. Does not test generalized sweet preference after sustained NNS exposure.
de Ruyter et al., 2013 [38]	203 children aged 7–11 y; double-blind RCT; 146 completed	250 mL/day sugar-free beverage sweetened with sucralose + acesulfame K vs. matched sucrose-sweetened beverage	18 months	Satiety; beverage liking; beverage wanting	No significant difference in satiety at 1 or 15 min. Children in the sugar group showed slightly greater liking and wanting of their assigned beverage (adjusted OR 1.63 and 1.65, respectively).	Strongest long-term pediatric trial directly assessing satiety. Liking/wanting concerned the study beverage and is not equivalent to generalized sweet preference.
de Ruyter et al., 2014 [45]	89 children aged 5–12 y; controlled sensory study	Seven NNS-sweetened beverages vs. matched sugar-sweetened versions	Single sensory session	Discrimination and immediate liking	Approximately 24% reliably discriminated NNS- from sugar-sweetened beverages; mean liking was identical for NNS and sugar versions (3.39 vs. 3.39; *p* = 0.9).	Direct evidence for immediate sensory acceptance, but not for changes in later preferred sweetness or food choice.
Rémy et al., 2014 [42]	44 children aged 8–11 y; within-subject conditioning study	Distinctly flavored high-energy sweet beverage (150 kcal) vs. no-energy sweet beverage	4-week conditioning, 3-week no-exposure period, 4-week reversed conditioning	Flavor liking; subsequent meal intake; caloric compensation	Both flavors initially increased in liking; after the no-exposure period the flavor previously paired with energy was liked more. Meal intake became lower after the high-energy beverage; compensation remained partial (29% after reversal).	Important evidence for flavor–energy learning and compensation, but the manipulation concerns flavor–energy pairing rather than an isolated effect of NNS.
van Engelen et al., 2014 [41]	15 normal-weight boys aged 9–14 y; randomized repeated-measures study	Noncaloric sucralose control vs. 200-kcal sucrose, HFCS or glucose solutions	Acute; beverage 60 min before ad libitum meal	Subjective appetite; subsequent food intake	Only glucose reduced subsequent food intake versus sucralose. Cumulative energy intake was higher after sucrose and HFCS than after sucralose.	Shows that results depend on the specific caloric comparator, not simply “NNS versus calories.” Very small, male-only sample.
Reigh et al., 2026 [43]	64 children aged 4–6 y; crossover study with five laboratory visits; mean age 5.9 y	NNS-sweetened apple juice, regular apple juice, apple slices, purée, or no preload	Acute crossover; visits ~1 week apart	Ad libitum meal intake; satiety/energy compensation	Relative to no preload, NNS-sweetened juice did not significantly reduce subsequent meal intake (estimate −5.4 kcal; *p* = 0.77), whereas regular apple juice did (−44.1 kcal; *p* = 0.03). Compensation also varied by sex across fruit-form conditions.	Particularly relevant new pediatric evidence, but the primary study question concerned food form; the NNS comparison was a secondary question.
Nakamura et al., 2026 [46]	128 children aged 8–13 y in the child consumer component	Lactose-hydrolyzed chocolate milks containing stevia/monk-fruit-based blends, including a blend containing allulose, vs. sucrose controls	Acute sensory acceptance testing	Overall liking and product acceptance	No significant differences in overall liking were observed between the tested sweetener-blend formulations and sucrose controls among child consumers.	Demonstrates that NNS-containing formulations can achieve comparable immediate acceptance. It does not assess learned preference or later food choice; some formulations also contained allulose, so the effect cannot be attributed to NNS alone.

Note: Studies may contribute to more than one behavioral domain when multiple outcomes were assessed within the same experiment. Multiple publications arising from the same underlying trial were not considered independent evidence. Immediate product liking or acceptance, learned flavor preference, preferred sweetness intensity, and subsequent food choice were treated as distinct outcomes. Anthropometric, metabolic, observational dietary-pattern, and prenatal-exposure studies were not included because these outcomes are not direct measures of appetite or food preference. No pediatric intervention study identified in this review directly examined whether sustained NNS exposure changes preferred sweetness intensity or subsequent habitual food choice.

## Data Availability

No new data were created or analyzed in this study. Data sharing is not applicable to this article.

## Abbreviations

AAP	American Academy of Pediatrics
Ace-K	acesulfame potassium
BMI	body mass index
CI	confidence interval
CPIR	cephalic-phase insulin response
ESPGHAN	European Society for Paediatric Gastroenterology, Hepatology and Nutrition
HFCS	high-fructose corn syrup
LCS	low-calorie sweeteners
NNS	nonnutritive sweeteners
NSS	non-sugar sweeteners
OR	odds ratio
RCT	randomized controlled trial
RR	risk ratio
SGLT1	sodium–glucose cotransporter 1
WHO	World Health Organization
